# Cardiovascular risk in patients with alpha-1-antitrypsin deficiency

**DOI:** 10.1186/s12931-017-0655-1

**Published:** 2017-09-15

**Authors:** Sebastian Fähndrich, Frank Biertz, Annika Karch, Björn Kleibrink, Armin Koch, Helmut Teschler, Tobias Welte, Hans-Ulrich Kauczor, Sabina Janciauskiene, Rudolf A. Jörres, Timm Greulich, Claus F. Vogelmeier, Robert Bals, on behalf of the COSYCONET investigators

**Affiliations:** 1 Department of Internal Medicine V, Pulmonology, Allergology, Intensive Care Medicine, Saarland University Hospital, 66424 Homburg, Germany; 2 Institute for Biostatistics, Hannover Medical School, 30625 Hannover, Germany; 3 Department of Pneumology, Ruhrlandklinik, West German Lung Center, and University Hospital Essen, University Duisburg-Essen, Essen, Germany; 4 Clinic for Pneumology, Hannover Medical School, Member of the German Center for Lung Research, 30625 Hannover, Germany; 5 Department of Diagnostic and Interventional Radiology, University of Heidelberg, 69120 Heidelberg, Germany; 6 Translational Lung Research Center (TLRC), Member of the German Center for Lung Research, Heidelberg, Germany; 7 Institute and Outpatient Clinic for Occupational, Social and Environmental Medicine, Ludwig-Maximilians-Universität München, 80336 Munich, Germany; 8 Department of Medicine, Pulmonary and Critical Care Medicine, University Medical Center Giessen and Marburg, Philipps-Universität Marburg, Marburg, Germany; 9 Member of the German Center for Lung Research (DZL), Marburg, Germany; 10 Department of Internal Medicine V – Pulmonology, Allergology, Intensive Care Medicine, 66421 Homburg, Saar Germany

**Keywords:** Alpha-1-antitrypsin deficiency, Phenotype, Cardiovascular disease, Emphysema, Personalized medicine

## Abstract

**Background:**

Alpha-1-antitrypsin deficiency (AATD) is a rare inherited condition caused by mutations of the SERPINA1 gene that is associated with the development of a COPD like lung disease. The comorbidities in patients with AATD-related lung diseases are not well defined. The aim of this study was to analyze the clinical phenotype of AATD patients within the German COPD cohort study COSYCONET (“COPD and SYstemic consequences-COmorbidities NETwork”) cohort focusing on the distribution of comorbidities.

**Method and results:**

The data from 2645 COSYCONET patients, including 139 AATD patients (110 with and 29 without augmentation therapy), were analyzed by descriptive statistics and regression analyses. We found significantly lower prevalence of cardiovascular comorbidities in AATD patients as compared to non-AATD COPD patients. After correction for age, pack years, body mass index, and sex, the differences were still significant for coronary artery disease (*p* = 0.002) and the prevalence of peripheral artery disease as determined by an ankle-brachial-index <= 0.9 (*p* = 0.035). Also the distribution of other comorbidities such as bronchiectasis differed between AATD and non-deficient COPD.

**Conclusion:**

AATD is associated with a lower prevalence of cardiovascular disease, the underlying mechanisms need further investigation.

**Electronic supplementary material:**

The online version of this article (10.1186/s12931-017-0655-1) contains supplementary material, which is available to authorized users.

## Background

Alpha-1-antitrypsin deficiency (AATD) is a rare inherited condition caused by mutations of the SERPINA1 gene and is a genetic risk factor for liver and lung disease [1, 2]. AATD is a model disease to explain the pathogenesis of chronic obstructive pulmonary disease (COPD) based on a dysbalance of proteases and antiproteases. COPD is a heterogeneous disorder with several phenotypes [3]. Comorbidities often complicate the clinical presentation of patients with COPD and comprise cardiovascular and cerebrovascular diseases, osteoporosis, depression, lung cancer, and diabetes [4]. Reduction in lung function is an independent risk factor for the presence of cardiovascular diseases [5] and the presence of cardiac disease impacts on the natural history of COPD and vice versa [6].

Only a few studies have investigated the comorbidity profile of AATD patients and found increased risk for aortic stiffness and musculoskeletal changes as compared to smokers without COPD [7] as well as a higher prevalence of bronchiectasis as compared to historical data [8]. Some studies reported that AATD patients without COPD have a lower risk for developing cardiovascular diseases as compared to PiMM individuals [9, 10], however this issue remains controversial [11, 12].

The aim of the present study was to analyze the clinical phenotype of AATD patients within the German COPD cohort “COPD and Systemic Consequences-Comorbidities Network” (COSYCONET). For this purpose, we identified AATD patients in the study cohort and analyzed their basic anthropometric data, pulmonary function parameters, and the presence of comorbidities with a focus on cardiovascular diseases.

## Methods

### Patient description

We used the baseline dataset of the COSYCONET cohort study (visit 1), which is a prospective, multicentre, observational study. In total, 2741 patients were recruited from September 2010 to December 2013 in 31 study centers throughout Germany. Patients were eligible if they were ≥40 years of age and had a diagnosis of COPD or symptoms of chronic bronchitis. Exclusion criteria were previous lung transplantation, lung volume reduction surgery and lung malignancies, and the presence of moderate or severe exacerbation within the last 4 weeks. Further details of the study have been reported elsewhere [13].

Patients were classified for COPD severity based on two different methods, GOLD criteria using the 2009 spirometric classification I-IV and the 2011 classification A-D based on the CAT score and spirometry (www.goldcopd.com). For the spirometric classification (GOLD 2009), patients showing a ratio of FEV_1_/FVC < 70% were allocated according their FEV_1_%pred and patients having a ratio of FEV_1_/FVC > 70% were classified as “at risk” for COPD or GOLD stage 0 [14] if they: (i) had a doctor’s diagnosis of chronic bronchitis, (ii) reported a severity of cough ≥3 points on the respective COPD Assessment Test (CAT) items and/or (iii) reported a severity of phlegm ≥3 points on the respective CAT item. The current analyses included all patients with GOLD stages 0-IV. Patients with missing severity stage (*n* = 19) and unclassified patients according to GOLD and the above definition (*n* = 77) were excluded from the analysis resulting in a sample of *n* = 2645 patients.

### Ethics, consent and permissions

The COSYCONET study was approved by the Ethics Committees of the local study centers. All cohort participants gave their written informed consent.

### Definition of alpha-1-antitrypsin deficiency (AATD)

The dataset of visit 1 comprised self-reported information on AATD, medication, and laboratory measurement of the serum AAT concentration. Genotyping data for the SERPINA1 gene were not available for most of the patients. Patients with known AATD were asked to specify this condition and the underlying genotype. Patients were assigned to two major groups: 1) COPD patients without AATD (“COPD”): Patients with no historical information of AATD, no augmentation therapy and normal AAT serum level. Patients with known heterozygous PiMZ status were classified as “COPD without AATD”.

2) Patients with (severe) AATD (“AATD”): Patients with known homozygous PiZZ or other homozygous deficiency mutations, patients with augmentation therapy. For missing genotypes, the medication and AAT serum concentration were evaluated: Subjects with AAT augmentation therapy with an AAT serum concentration value <50 mg/dl were classified as “COPD with AATD”. Based on the presence of augmentation therapy, the “AATD” group was subdivided into AATD with augmentation therapy (“AATD + T”) and without (“AATD-T”). Medication lists were screened for all patients and presence of augmentation therapy led to the classification as AATD patient.

### Measurements

Clinical history including information on smoking habits and comorbidities was accessed during visit 1. A 6-min walk test (6MWT) was performed according to the 2002 ATS guidelines [15]. The BODE index was computed using the algorithm developed by Celli and colleagues [16]. For the time-up-and-go-test, the duration of the procedure (stand up from the sitting position, walk of 3 m distance and back and sit down again) was measured [17]. Pulmonary function testing by spirometry and body plethysmography was performed according to the ATS/ERS guidelines [18] and reference equations were used as described by the Global Lung Function Initiative (GLI) [19]. The diffusing capacity for carbon monoxide (TLCO) was determined by the single-breath technique following the ERS/ATS guidelines [20]. The ankle-brachial Index, ABI) was measures as described [21] and an ABI of <0.9 was considered abnormal according to the American College of Cardiology Foundation/American Heart Association Task Force Practice Guidelines [22]. Within COSYCONET, patients were asked to make their CT scans available for analysis. These were visually assessed for emphysema and airway changes by a senior radiologist with more than 15 years of experience in chest imaging and COPD. In a lobe-based approach, emphysema was rated semi-quantitatively on a five-point scale for each lobe as follows: <5%, 5–25%, 26–50%, 51–75%, >75%. The emphysema was classified as being centrilobular, including coalescent centrilobular emphysema or panlobular, including advanced destructive emphysema [23]. Large airways were assessed for the presence of bronchiectasis and wall thickening, small airways for centrilobular nodules and mosaic attenuation pattern. Finally, a decision was made whether the predominant component of the disease was emphysema or abnormalities of the airways.

### Statistical analysis

The statistical analysis was performed using SAS software version 9.3 (SAS Institute Inc., Cary, NC, USA). *P*-values ≤0.05 were considered statistically significant. For quantitative variables, results are shown as lsmean, difference between lsmeans with CI 95% and *p*-values from GLM (if not stated otherwise). The General Linear Models (GLM) procedure is a method of linear regression that uses the method of least squares and is more robust for variables that do not show a normal distribution. Least squares means (lsmean) refers to as marginal means (or sometimes EMM - estimated marginal means). In an analysis of covariance model, they are equivalent to the group means after having controlled for a covariate. Categorical variables are reported as absolute and relative (%) frequencies and chi-square tests were performed to compare groups. Results are visualized using (grouped) bar charts, histograms or forest plots.

In order to adjust the comparisons for potential confounders, multivariate regression analyses and subgroup analyses were conducted. Comorbidities were compared using logistic regression models adjusted for sex, age groups, pack years, FEV1% pred (GLI), BMI and hypertension (yes/no). Sensitivity analyses omitting hypertension as influencing factor were performed. A Forest plot was used to visualize the adjusted odds ratios (ORs) of selected comorbidities with corresponding 95%-confidence intervals. Differences regarding laboratory parameters were analyzed with linear regression adjusted for sex, age groups, pack years, FEV1% predicted and BMI.

An overall sensitivity analysis was applied in a selected dataset matching 3 non-AATD patients to each AATD patient. Matching criteria were sex, age (± 5 years) and pack years (± 10 years). For all AATD patients but one, 3 controls could be found. Comparisons in this dataset were performed in line with the analysis strategy outlined above.

## Results

### Patient characteristics

A total of 139 patients with AATD were identified (5.2% of total cohort). Of these, 110 patients (79.1%) received AAT augmentation therapy, whereas 29 patients (20.9%) reported no augmentation therapy or information on this therapy was missing. The AATD group comprised 106 Pi ZZ patients (78.5%), 2 with PiSZ genotype, 20 with unknown genotype but augmentation therapy and 11 patients with unknown genotype and no or unknown augmentation treatment. These 11 patients were classified as AATD patients because of the low serum levels of AAT (< 50 mg/dl). The group of COPD subjects without AATD included 8 patients with self-reported AATD of genotype Pi MZ, 16 patients with self-reported AATD of unknown genotype but normal AAT serum level and no replacement treatment, and 2482 patients without AATD according to self-report and medication.

The characteristics of the individuals with AATD with (“AATD + T”) or without augmentation therapy (“AATD-T”) and individuals with COPD only (“COPD”) are shown in Table 1. As expected, there were significant differences between COPD and AATD patients. AATD patients were younger, had a lower BMI, and had smoked considerably less. AATD-T as compared to AAT + T patients were younger, showed less severe lung disease and better performance in the 6-MWT.

**Table 1:** Comparison of patient characteristics of the analysis population

	Total 2645	1 AATD + T (110)	2 AATD-T (29)	3 COPD (2506)	Diff. between means (CI95%) *p*-value
Male sex	1572 (59.4%)	67 (60.9%)	10 (34.5%)	1495 (59.7%)	1 vs. 3: 1.05 (0.71 1.56) 0.791 ~ 2 vs. 3: 0.36 (0.16 0.77) < .01
Age (years)	65.00 (64.67–65.33)	59.47 (57.88–61.07)	61.28 (58.18–64.38)	65.29 (64.95–65.62)	1–2: −1.80 (−5.28–5.29) 0.310 1–3: −5.81 (−7.44–4.19) < .0001 2–3: −4.02 (−7.13–0.90) 0.012
COPD duration (years)	7.80 (7.53–8.06)	11.24 (9.95–12.53)	9.57 (7.02–12.13)	7.63 (7.35–7.90)	1–2: 1.67 (−1.19 4.52) 0.254 1–3: 3.61 (2.29 4.92) < .0001 2–3: 1.95 (−0.63 4.51) 0.138
Weight (kg)	78.91 (78.22–79.59)	75.11 (71.75–78.47)	74.28 (67.73–80.83)	79.13 (78.43–79.83)	1–2: 0.83 (−6.53 8.19) 0.824 1–3: −4.02 (−7.46–0.59)0.022 2–3: −4.86 (−11.5 1.73) 0.149
BMI	26.97 (26.76–27.17)	24.59 (23.59–25.59)	24.76 (22.81–26.71)	27.10 (26.89–27.31)	1–2: −0.17 (−2.36 2.02) 0.881 1–3: −2.51 (−3.53–1.48) < .0001 2–3: −2.34 (−4,30–038) 0.019
Packyears	44.31 (42.91–45.72)	16.3 (9.54–23.04)	11.8 (−1.37 24.91)	45.9 (44.52–47.35)	1–2: 4.52 (−10.26 19.29) 0.549 1–3: −29.6 (−36.54–22.75) < .0001 2–3: −34.16 (−47.38–20.94) < .0001
SGRQ Score	43.05 (42.28–43.81)	45.86 (42.13–49.59)	34.45 (27.18–41.71)	43.02 (42.24–43.81)	1–2: 11.41 (3.25 19.58) 0.006 1–3: 2.82 (−099 6.62) 0.148 2–3: −8.59 (−15.90–1.29) 0.021
CAT	18.00 (17.72–18.28)	18.79 (17.41–20.17)	16.59 (13.92–19.25)	18.15 (17.86–18.44)	1–2: 2.20 (−0.80 5.20) 0.151 1–3: 0.63 (−0.78 2.04) 0.382 2–3: −1.57 (−4.25 1.11) 0.251
EQ5D	0.82 (0.81–0.83)	0.83 (0.79–0.87)	0.87 (0.80–0.95)	0.82 (0.81–0.82)	1–2: −0.04 (−0.13 0.04) 0.324 1–3: 0.01 (−0.03 0.06) 0.488 2–3: 0.06 (−0.02 0.13) 0.142
6-min-walk distance (m)	419.29 (415.10–423.48)	421.86 (400.99–442.74)	495.96 (455.92–536.01)	418.30 (414.01–422.59)	1–2: −74.1 (−119.3–28.9) 0.001 1–3: 3.6 (−17.7 24.9) 0.736 2–3: 77.7 (37.5118.0) 0.0002
Time-up-and-go (seconds)	6.99 (6.90–7.08)	6.22 (5.76–6.689	6.44 (5.53–7.34)	7.03 (6.93–7.12)	1–2: −0.21 (−1.23 0.80) 0.681 1–3: −0.81 (−1.27–0.34) < .001 2–3: −0.59 (−1.51 0.32) 0.204
BODE Score	2.29 (2.21–2.37)	2.90 (2.52–3.29)	1.29 (0.55–2.02)	2.27 (2.19–2.35)	1–2: 1.62 (0.79 2.45) < .001 1–3: 0.63 (0.23 1.02) < .01 2–3: −0.99 (−1.73–0.25) < .01

~ point estimate (CI95%) Chi-Square *p*-value

We next focused on the lung disease phenotype (Table 2). As compared to non-deficient COPD, AATD + T patients showed more severe airflow limitation as indicated by significantly lower FEV_1_%pred, lower FEV_1_/FVC and increased effective specific airway resistance sRaweff %pred. In contrast, AATD-T individuals had less airflow limitation as compared to the COPD or AATD + T groups. Some patients provided CT scans obtained for the clinical purposes up to 4 years prior to inclusion into our study. In total, 356 CT scans from 2506 COPD patients (14.2%) and 23 CT scans from 139 AATD patients (16.5%) were available, which did not allow to perform AATD patients grouping according to augmentation therapy. Hence, AATD patients showed more emphysema, with 16% having an involvement of more than 75% of lung as compared to only 3% within COPD group (Additional file 1: Table S1). At the same time, wall thickening of the airways was less frequently observed in the AATD group, while bronchiectasis was more frequent as compared to the COPD group.

**Table 2:** Pulmonary function measurements reveal difference between AATD-COPD and COPD

	Total (2645)	1 AATD + T (110)	2 AATD- T (29)	3 COPD (2506)	Diff. between means (CI 95%) *p*-value
FEV1 (L)	1.65 (1.63–1.68)	1.46 (1.32–1.59)	2.11 (1.85–2.37)	1.66 (1.63–1.68)	1–2: −0.65 (−094–0.36) < .0001 1–3: −0.20 (−0.33–0.06) < .001 2–3: 0.45 (0.19 0.71) < .001
FEV1% predicted	56.28 (55.84–57.08)	45.14 (41.25–49.02)	68.86 (61.29–76.43)	56.62 (55.81–57.44)	1–2: −23.72 (−32.2–15.2) < .0001 1–3: −11.50 (−15.4–7.5) < .0001 2–3: 12.3 (4.6 19.9) < .001
FVC % predicted	78.55 (77.82–79.28)	76.00 (72.42–79.55)	85.70 (78.76–92.64)	78.58 (77.83–79.33)	1–2: −9.71 (−17.5–1.9) 0.014 1–3: −2.58 (−6.22 1.06) 0.164 2–3: 7.12 (0.14 14.10) 0.046
FEV1/VC	0.55 (0.54–0.55)	0.46 (0.44–0.49)	0.61 (0.56–0.66)	0.55 (0.55–0.56)	1–2: −0.15 (−0.20–0.09) < .0001 1–3: −0.09 (−0.11–0.06) < .0001 2–3: 0.06 (0.01 0.10) 0.020
RV (L)	3.82 (3.77–3.87)	4.49 (4.26–4.7)	3.70 (3.26–4.15)	3.79 (3.75–3.84)	1–2: 0.79 (0.29 1.29) 0.002 1–3: 0.69 (0.46 0.93) < .0001 2–3: −0.09 (−0.54 0.35) 0.685
RV/ TLC nominal value (%)	134.10 (133.00–135.29)	145.53 (140.15–150.91)	127.76 (117.43–138.08)	133.67 (132.54–134.80)	1–2: 17.77 (6.12 29.42) 0.003 1–3: 11.85 (6.35 17.35) < .0001 2–3: −5.91 (−16.31 4.48) 0.265
ITGV % predicted	145.13 (143.69–146.58)	172.25 (165.29–179.22)	148.02 (134.58–161.47)	143.90 (142.43–145.36)	1–2: 24.23 (9.09 39.38) 0.002 1–3: 28.35 (21.23 35.47) < .0001 2–3: 4.12 (−9.41 17.64) 0.550
TLC (L)	7.13 (7.07–7.18)	8.18 (8.00–8.46)	7.46 (6.91–8.00)	7.08 (7.02–7.14)	1–2: 0.72 (0.11 1.33) 0.021 1–3: 1.11 (0.82 1.40) < .0001 2–3: 0.39 (−0.16 0.93) 0.165
sRaw eff % predicted	170.90 (165.81–176.00)	179.28 (154.29–204.27)	120.75 (73.38–168.13)	171.14 (165.91–176.37)	1–2: 58.53 (4.94112.11) 0.032 1–3: 8.11 (−17.43 33.65) 0.534 2–3: −50.42 (−98.10–2.74) 0.038
TLCO % predicted	55.01 (54.17–55.86)	43.92 (39.79–48.05)	56.49 (48.71–64.27)	55.47 (54.61–56.33)	1–2: −12.57 (−21.38–3.76) 0.005 1–3: −11.54 (− 15.77–7.33) < .0001 2–3: 1.02 (−6.82 8.85) 0.799

**Fig. 1:**
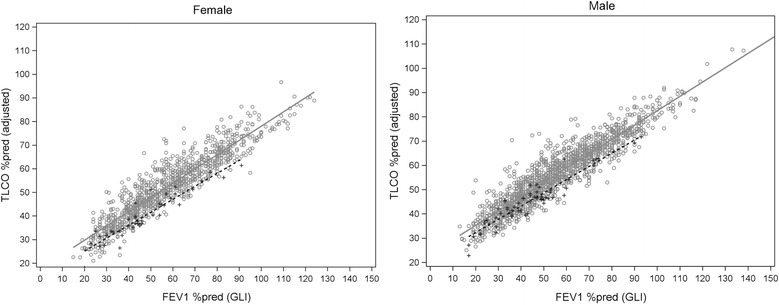
The presence of AATD in males and female patients (broken line) is associated with a significant reduction of TLCO %pred as compared to patients with COPD (straight line). The multivariate analysis included adjustment for airway obstruction in terms of FEV_1_% pred, lung hyperinflation in terms of ITGV %pred and COPD risk factors in terms of packyears and BMI

Next we performed a multivariate regression analysis with TLCO %pred as dependent variable, which is related to emphysema and linked to other parameters that differ between AATD and COPD groups [24]. The presence of AATD was associated with a significant reduction of TLCO %pred after adjustment for airway obstruction in terms of FEV1% pred, lung hyperinflation in terms of ITGV %pred and for COPD risk factors, such as smoking (pack-years) and BM (Fig. 1 and Additional file 1: Table S2). This difference is illustrated for ITGV%pred in Additional file 1: Figure S1A, B and for BMI in Additional file 1: Figure S1C, D. The reduction of TLCO in AATD is greater than expected from other parameters known to be associated with COPD and emphysema.

Sensitivity analysis in a dataset matched for age, sex and pack years was performed. In the matched dataset, AATD-COPD patients (*n* = 138) had a similar 6-min walk distance and a comparable time-up-and-go test as patients of the COPD group (*n* = 414) (6-MWT: 437 ± 111 vs. 440 ± 11 m, time-up-and-go: 6.0 vs. 6.2 s as median time). Differences in pulmonary assessments remained highly significant in the matched dataset. sRaweff showed a (nearly significant) trend towards increased airflow resistance in the AATD-COPD patients with a median of 133%pred. as compared to 115%pred. in patients without AATD (*p* = 0.053).

### Cardiovascular diseases are less frequent in AATD + T patients after adjustment for potential confounders

**Fig. 2:**
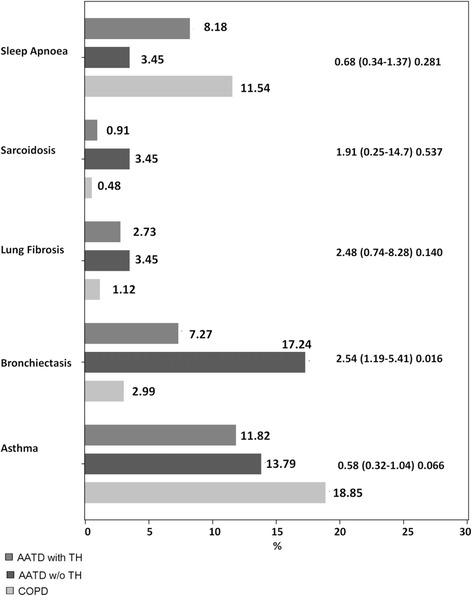
Frequency of self-reported pulmonary comorbidities in patients with COPD, AATD + T and AATD-T (OR (CI95%) *p*-value, compared AATD + T vs. COPD); TH = augmentation therapy

In a first step, we analyzed lung-specific comorbid entities, for which AATD individuals reported increased prevalence of bronchiectasis as compared to the COPD patients (Fig. 2).

**Fig. 3:**
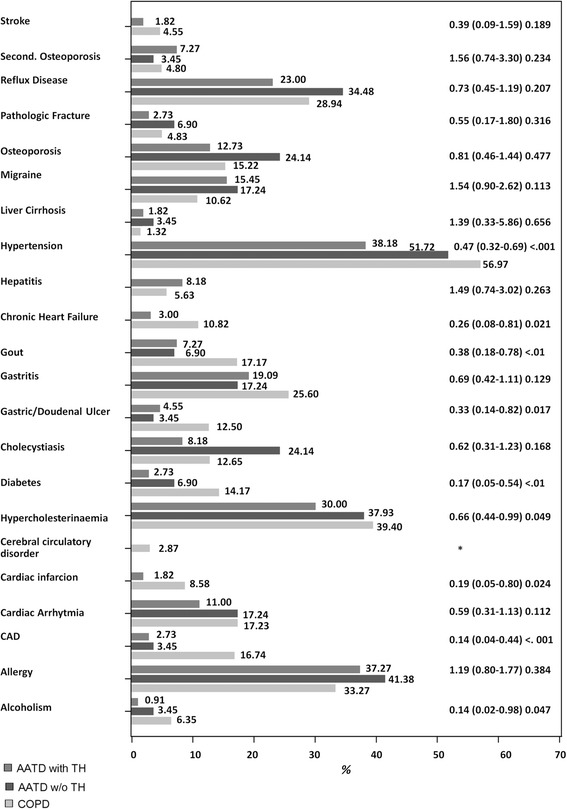
Distribution of extrapulmonal comorbidities in patients with COPD, AATD + T and AATD-T (OR (CI95%) *p*-value, compared AATD + T vs. COPD)

**Fig. 4:**
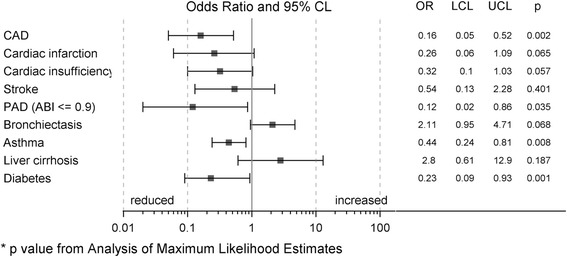
Distribution of comorbidities in AAT-COPD patients: Forest plot for the adjusted influence of AATD (yes/no) on different comorbidities (odds ratios are derived from multivariable logistic regression models)

Next, we analyzed the distribution of extra-pulmonary comorbidities and found significant differences between AATD and COPD patients, with particularly lower frequencies of cardiovascular and related diseases (hypertension, chronic heart failure, diabetes, cardiac infarction, and cardiac arterial disease) in AATD (Fig. 3). These changes were concordant for patients with and without augmentation therapy and remained significant when AATD + T and AATD-T were combined (prevalence in COPD vs. AATD: hypertension 57 vs. 41%, chronic heart failure 11 vs. 2%, diabetes without insulin 9 vs. 3%, cardiac infarction 9 vs. 1%, and cardiac arterial disease 3 vs. 17%). Logistic regression models with adjustment for sex, age, pack years, FEV_1_%pred, BMI and hypertension revealed significant differences between CODP and AATD + T patients for selected comorbidities (Fig. 4 and Additional file 1: Table S3). The number of AATD-T patients was too low to perform this type of analysis. These data indicate that in AATD + T patients cardiovascular diseases were less frequent after adjustment for potential confounders. Based on the small number of patients without augmentation, no conclusion can be drawn about the effect of therapy.

### AATD is associated with lower triglyceride and lower HbA1c levels

To reveal whether AATD patients have altered levels of biochemical risk markers for cardiovascular disease in the blood, we analyzed laboratory data (Table 3). Levels of triglycerides were lower in the AATD + T group, while cholesterol was higher in the AATD-T group. HDL-cholesterol was higher in AATD patients independent of augmentation therapy. HbA1c was lower in AATD patients. As compared to the COPD group, HDL-cholesterol was higher and HbA1c was lower in AATD patients independent of augmentation therapy. Multivariate linear analyses adjusting for sex, age, packyears, FEV_1_%pred and BMI supported these findings (parameter estimates, *p*-values): triglycerides as well as HBa1c were lower in AATD patients as compared to COPD (triglycerides −39.5 mg/dl, *p* < 0.001; HBa1c −2.5 mmol/mol, *p* = 0.002). HDL-cholesterol was not significantly different between AATD + T and AATD-T (+2.4, *p* = 0.223), but cholesterol was significantly higher in AATD-T group (+22.9, *p* = 0.038).

**Table 3:** Comparison of laboratory investigations in patients with COPD and AATD (with and without augmentation therapy). For all laboratory parameters, lsmean, diff-lsmean and CL95% and *p*-value from GLM are reported if not otherwise stated

Item	Total ^a^ (*n* = 2645)	1 AATD + T (*n* = 110)	2 AATD-T (*n* = 29)	3 COPD (*n* = 2506)	Diff. between means (CI 95%) *p*-value
Creatinine serum [mg/dl]	0.89 (0.88–0.90)	0.86 (0.81–0.91)	0.75 (0.66–0.85)	0.89 (0.88–0.89)	1–2: 0.11 (−0.001 0.22) 0.053 1–3: −0.029 (−0.08 0.20) 0.243 2–3: −0.14 (−0.24–0.04) < .01
Uric acid [mg/dl]	5.95 (5.88–6.01)	5.5 (5.2–5.8)	5.2 (4.6–5.9)	5.9 (5.9–6.0)	1–2: 0.23 (−0.46 0.94): 0.509 1–3: −0.51 (−0.83–0.18) 0.002 2–3: −0.75 (−1.38–0.11) 0.021
alpha-1-antitrypsin [mg/dl]	142 (141–143)	113 (107–119)	39 (28–51)	144 (143–146)	1–2: 73.49 (60.46 86.51) < .0001 1–3: −31.94 (−38.07–25.82) < .0001 2–3: −105.43 (−117.05–93.81) < .0001
Triglyceride [mg/dl]	142 (138–146)	94 (73–116)	135 (93–178)	144 (140–149)	1–2: −41.02 (−88.55 6.51) 0.091 1–3: −50.56 (−72.35–28.76) < .0001 2–3: −9.53 (−52.24 33.17) 0.662
Cholesterol [mg/dl]	216 (215–217)	217 (209–225)	245 (229–261)	216 (214–218)	1–2: −27.91 (−46.38–9.44) 0.003 1–3: 1.27 (−7.17 9.71) 0.767 2–3: 29.18 (12.58 45.79) < .001
HDL Choles-terol [mg/dl]	65 (64–66)	71 (67–76)	75 (66–83)	64 (64–65)	1–2: −3.27 (−12.62 6.08) 0.491 1–3: 7.01 (2.69 11.32) 0.002 2–3: 10.27 (1.89 18.66) 0.016
LDL Choles-terol [mg/dl]	128 (127–130)	127 (120–135)	144 (130–159)	128 (126–129)	1–2: −17.15 (−33.70–0.60) 0.042 1–3: −0.57 (−8.21 7.07) 0.884 2–3: 16.58 (1.74 31.41) 0.029
HBA1c [mmol/mol]	41.2 (40.8–41.5)	37.1 (35.6–38.6)	37.1 (34.2–39.9)	41.4 (41.1–41.7)	1–2: 0.01 (−3.25 3.28) 0.994 1–3: −4.29 (−5.84–2.74) < .0001 2–3: −4.30 (−7.21–1.39) 0.004

^a^Mean CL95% reported

## Discussion

The main finding of the present study is that AATD-related lung disease is associated with fewer manifestations of periphery and coronary artery disease after correction for smoking, age and other potential confounders.

Non-deficient COPD is associated with comorbidities such as cardiovascular disease, lung cancer, or osteoporosis [25, 26]. A recent study based on health insurance data found a decreased prevalence of ischemic heart disease in AATD individuals as compared to COPD patients [27]. In the present study, hypertension, diabetes, coronary artery disease, heart failure, and alcoholism were reported significantly less often in AATD as compared to non-AATD COPD. The observed differences in comorbidities between AATD-COPD and COPD patients are associated with biochemical markers, such as significantly lower triglyceride concentrations and lower HbA1c in AATD-COPD than in COPD. After correction for potential cofounders such as age, smoking history and BMI, cardiovascular diseases were still found less frequent among AATD-COPD as compared to COPD patients. The observed differences in the prevalence of hypercholesterolemia between the self-reported data (Fig. 3) and the laboratory measurements (Table 3) could results from the different data sourced or the effect of cholesterol-lowering therapies.

This finding highlights the possibility of specific mechanisms in AATD and/or augmentation therapy that may interfere with the development of cardiovascular disease. There are several contradicting studies that highlight this potential link: AATD patients have increased aortic stiffness compared to control individuals without COPD, as determined by aPWV [7]. This finding was replicated in another study [28]. Earlier studies also observed a reduced blood pressure in AATD [9], however others authors did not find such differences [29]. A genetic study in the Copenhagen City Heart cohort revealed that the systolic blood pressure is lower in PiZZ and PiMZ individuals compared to PiMM or PiMS individuals. However, PiMZ heterozygosity was associated with increased age as a potential confounder [10]. A genetic association study examined the frequency of AATD mutations in patients with coronary atherosclerosis and healthy controls and found an association of heterozygosity in the patient group [11]. As to our knowledge, the present study is the first analysis of a direct comparison of non-deficient and AATD-based COPD patients. It is important to point out that the association of AATD with reduced frequencies of hypertension and ischemic heart disease, or a low ABI (as marker of peripheral artery disease) could only be demonstrated for the combined AATD-T and AATD + T group of patients. The low number of patients within AATD-T subgroup (only 29 patients) did not allow a separate statistical evaluation. Similarly, a previous study based on insurance data [27] had no information on augmentation therapy. Thus, we are not able to make a firm conclusion whether our finding is associated with AATD per se and/or with augmentation therapy. Based on the small number of patients without augmentation, no conclusion can be drawn about the effect of therapy.

The mechanisms that link AATD or augmentation therapy with decreased frequency of cardiovascular disease are speculative and may be related to the pleiotropic activities of AAT [30, 31] These activities might include i) loss of vascular elastic recoil and decreased resistance due to excess activity of elastase; ii) upregulation and release of angiopoietin-like protein 4 (Angptl4) by AAT in complex with fatty acids [32–34].; iii) decreased production of inflammatory cytokines, such as TNF-α and IL-1β by AAT [35]. In addition, a protective role for AAT was demonstrated in the Lipid Coronary Angiography Trial that evaluated male participants after coronary bypass surgery [12]. Altogether, the data above suggest that the mechanisms that links AATD or augmentation therapy with decreased frequency of cardiovascular disease are likely related to the pleiotropic activities of AAT protein. The effect of AATD on cardiovascular risk might represent an advantageous consequence of the SERPINA1 mutation, in addition to a proposed selective anti-infective advantage by increased inflammation [36].

The present study revealed additional characteristics of AATD-related lung disease. Patients with AATD significantly more often reported the presence of bronchiectasis, which is in line with previous data [8]. Another study analyzed the distribution of AATD alleles among patients with bronchiectasis and found an even distribution between patients and controls [37]. The analysis of the lung phenotype revealed an out-of-proportion loss of diffusion capacity. AATD lung disease is associated with panlobular emphysema, with a predominance in the lower lobe, and with a loss of elastic recoil pressure [38, 39]. CT studies have shown heterogeneity of the distribution of emphysema [40, 41]. Indeed, AATD was associated with a significant reduction of TLCO %pred after adjustment for ITGV%pred as well as FEV_1_%pred, packyears and BMI as other potentially relevant confounders. A defect of diffusion capacity is known to associate with worse quality of life [41] and a decrease of KCO was associated with apical loss of lung parenchyma in CT [40].

Several limitations of the present study have to be taken into account: In the present study the information on comorbidities was based on self-reported statements of a doctor’s diagnosis. Although the COSYCONET cohort study is a multicenter study with a large number of patients, the number of AATD individuals with or without augmentation therapy is limited. The low number of patients without therapy made it difficult to discriminate whether the effect of AATD on cardiovascular risk is associated with the disease or with augmentation therapy. Genotyping data on the SERPINA1 gene were only available for a minority of the patients. Nevertheless, the applied grouping algorithm likely results in a correct separation of individuals with severe AATD. The recruitment strategy of COPD and AATD patients was likely different and could account for a selection and confounding bias. Data on the time course of augmentation therapy were not available.

## Conclusion

In conclusion, within the German COPD cohort COSYCONET we found that AATD is associated with a lower number of cardiovascular comorbidities even after adjustment for confounders. The underlying mechanisms are currently unclear. These data add to the understanding of the complex biology of AATD and indicated that AATD likely impacts on processes involved in cardiovascular disease.

## Additional file

Additional file 1: Table S1. Analysis of lung density by CT (available for 379 patients). (f) *n* = 3 missings, (g) *n* = 89 missings, *panlobular emphysema, PLE; centrilobular emphysema, CLE. **Table E2.** Multivariate linear regression model with TLCO %pred. as dependent variable. AATD refers to the presence of AATD versus COPD. **Figure S1.** Patients with AATD revealed a reduced TLCO %pred at similar levels of ITGV %pred. (A, B) or BMI (B, D) for both female and male patients after adjustment for FEV1, sex, BMI, packyears, and age (AATD - dotted line, COPD - straight line). **Table S3.** Results (Odds Ratio [95% confidence interval] and *p*-value) of multivariable logistic regression analyses for different comorbidities as dependent variable. (DOCX 265 kb)

## Abbreviations

6-MWT: 6-min walk test
ABI: Ankle-brachial-index
alpha-1: Antitrypsin deficiency
BMI: Body mass index
CAT: COPD assessment test
COPD: Chronic obstructive lung disease
COSYCONET: COPD and SYstemic consequences-COmorbidities NETwork
FEV_1_: Forced expiratory volume in 1 s
FVC: Forced vital capacity
OR: Odd ratio
sRaw: Specific airway resistance
TLCO: Transfer factor for CO
