# Supplementary material for: Cardiovascular risk in patients with alpha-1-antitrypsin deficiency
Source: Respir Res. 2017 Sep 15;18:171. doi: 10.1186/s12931-017-0655-1 (PMC5602961; doi:10.1186/s12931-017-0655-1)
Supplement: Additional file 1: Table S1. — Analysis of lung density by CT (available for 379 patients). (f) n = 3 missings, (g) n = 89 missings, *panlobular emphysema, PLE; centrilobular emphysema, CLE. Table E2. Multivariate linear regression model with TLCO %pred. as dependent variable. AATD refers to the presence of AATD versus COPD. Figure S1. Patients with AATD revealed a reduced TLCO %pred at similar levels of ITGV %pred. (A, B) or BMI (B, D) for both female and male patients after adjustment for FEV1, sex, BMI, packyears, and age (AATD - dotted line, COPD - straight line). Table S3. Results (Odds Ratio [95% confidence interval] and p-value) of multivariable logistic regression analyses for different comorbidities as dependent variable. (DOCX 265 kb) [file 12931_2017_655_MOESM1_ESM.docx]

**Patients with alpha-1-antitrypsin deficiency show decreased cardiovascular risk and out-of-proportion reduction of CO diffusing capacity**

Sebastian Fähndrich^1^, Frank Biertz^2^, Annika Karch^2^, Björn Kleibrink^3^, Armin Koch^2^, Helmut Teschler^3^, Tobias Welte^4^, Hans-Ulrich Kauczor^5^, Sabina Janciauskiene^4^, Rudolf A. Jörres^6^, Timm Greulich^7^, Claus F. Vogelmeier^7^, Robert Bals^1^ on behalf of the COSYCONET investigators

**Online Supplement**

Table E1: Analysis of lung density by CT (available for 379 patients). (f) n=3 missings, (g) n=89 missings, *panlobular emphysema, PLE; centrilobular emphysema, CLE

|  | **Total**  **(n=2645)** | **COPD**  **(n=2506)** | **AATD-COPD**  **(n=139)** | **p-value** |
| --- | --- | --- | --- | --- |
| Emphysema predominance ^(f)^  Emphysema type CLE (%)*  Emphysema type PLE (%)* | 200 (53.2%)  80.3  19.7 | 182 (51.6%)  82.9  17.1 | 18 (78.3%)  49.2  50.8 | 0.129  <.0001 |
| Bronchiectasis Yes (%) | 9.6 | 8.1 | 32.6 | <.0001 |
| Wall thickening Yes (%) | 58.5 | 60.2 | 33.3 | <.0001 |
| Lung density percentile 15 (Hounsfield Units) ^(g)^ | -942 ± 34 | -941 ± 34 | -966 ± 29 | 0.002 |
| Emphysema index (%) ^(g)^ | 17.9 ± 16.0 | 17.0 ± 15.5 | 31.7 ± 18.5 | <.001 |

Table E2: Multivariate linear regression model with TLCO %pred. as dependent variable. AATD refers to the presence of AATD versus COPD.

| Variable | | Parameter estimate | Standard error | p-value |
| --- | --- | --- | --- | --- |
| Intercept | | 25.71945 | 4.56961 | <0.0001 |
| FEV1 %pred (GLI) | | 0.51612 | 0.01976 | <0.0001 |
| Sex | | -5.89228 | 0.71373 | <0.0001 |
| AATD | | -4.93557 | 1.53956 | 0.0014 |
| BMI (kg/m²) | | 0.75508 | 0.06783 | <0.0001 |
| Packyears [Y] | | -0.06259 | 0.00950 | <0.0001 |
| Age [Y] |  | 0.11015 | 0.03996 | 0.0059 |
| ITGV %pred | | -0.02674 | 0.01184 | 0.0241 |

Figure E1: Patients with AATD revealed a reduced TLCO %pred at similar levels of ITGV %pred. (A, B) or BMI (B, D) for both female and male patients after adjustment for FEV1, sex, BMI, packyears, and age (AATD - dotted line, COPD - straight line).


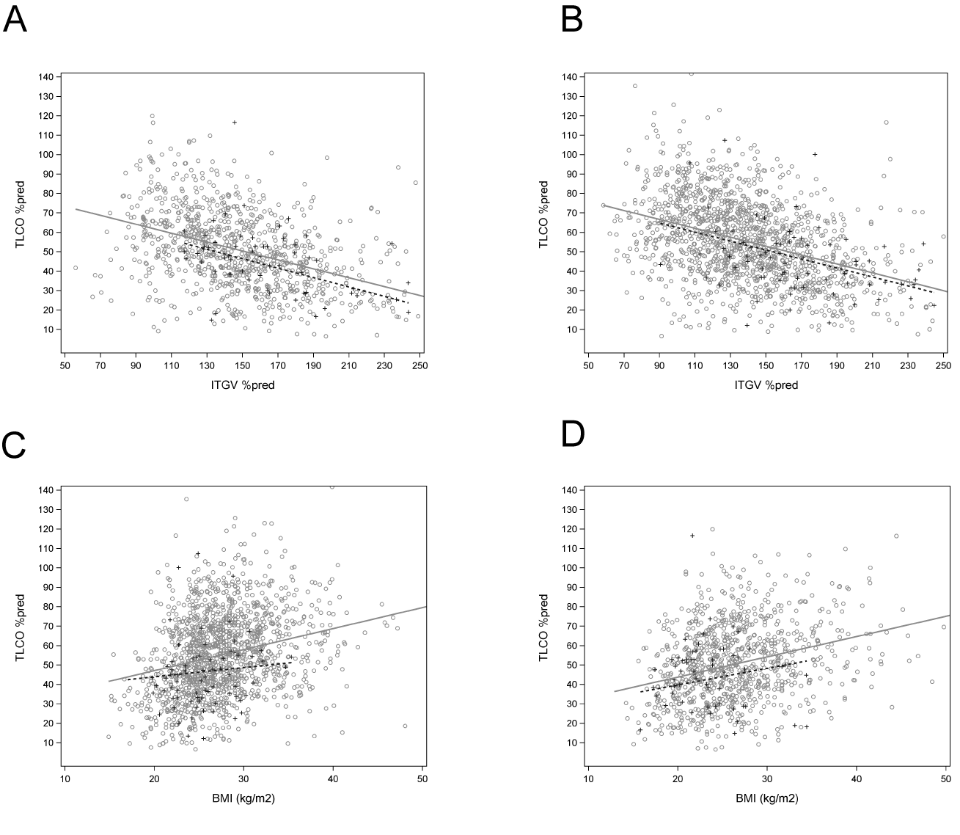


Table E3: Results (Odds Ratio [95% confidence interval] and p-value) of multivariable logistic regression analyses for different comorbidities as dependent variable

|  | **AATD with therapy vs. COPD** | **Sex** | **Age group** | **Packyears** | **FEV1 %predicted** | **BMI** | **Hypertension** yes/no |
| --- | --- | --- | --- | --- | --- | --- | --- |
|  |  | f/m | (≥65)/(<65) | (years) | (%) |  |  |
| **Bronchiectasis** | **2.11** [0.95-4.71] | 0.69 [0.43-1.11] | 1.78 [1.11-2.86] | 0.99 [0.989-1.01] | 1.0 [0.99-1.12] | 0.97 [0.92-1.02] | 0.73 [0.46-1.12] |
|  | 0.068 | 0.129 | 0.017 | 0.027 | 0.916 | 0.170 | 0.174 |
| **Asthma** | **0.44** [0.24-0.81] | 1.66 [1.35-2.04] | 0.82 [0.67-1.00] | 0.99 [0.985-0.992] | 1.02 [0.997-1.01] | 1.18 [0.99-1.04] | 0.92 [0.74- 1.14] |
|  | 0.008 | <.0001 | 0.055 | <.0001 | 0.450 | 0.071 | 0.433 |
| **Coronary Artery Disease** | **0.16** [0.05-0.52] | 0.38 [0.29-0.49] | 1.75 [1.38-2.21] | 0.99 [0.996-1.002] | 0.99 [0.988-0.999] | 1.03 [1.01-1.05] | 2.68 [2.07-3.46] |
|  | 0.002 | <.0001 | <.0001 | 0.391 | 0.014 | 0.004 | <.0001 |
| **Peripheral Artery Disease (ABI ≤ 0.9)** | **0.12** [0.02-0.86] | 0.76 [0.55-1.04] | 2.32 [1.67-3.22] | 1.00 [0.998-1.006] | 0.98 [0.974-0.990] | 0.99 [0.96-1.02] | 1.92 [1.38-2.68] |
|  | 0.035 | 0.087 | <.0001 | 0.353 | <.0001 | 0.458 | <.001 |
| **Stroke** | **0.54** [0.13-2.28] | 0.81 [0.54-1.23] | 1.61 [1.06-2.42] | 1.00 [0.998-1.008] | 1.00 [0.99-1.01] | 0.99 [0.96-1.02] | 2.13 [1.48-3.62] |
|  | 0.401 | 0.329 | 0.024 | 0.240 | 0.933 | 0.916 | <.001 |
| **Liver cirrhosis** | 2.8 [0.61-12.9] | 0.92 [0.45-1.87] | 1.21 [0.61-2.43] | 1.00 [0.995-1.012] | 1.01 [0.99-1.03] | 1.10 [1.05-1.16] | 1.54 [0.71-3.31] |
|  | 0.187 | 0.812 | 0.588 | 0.382 | 0.274 | <.001 | 0.269 |
